# High levels of fecal calprotectin and C-reactive protein in patients with colitis

**DOI:** 10.25122/jml-2021-0311

**Published:** 2023-01

**Authors:** Brinna Anindita, Titong Sugihartono, Muhammad Miftahussurur, Ummi Maimunah, Iswan Abbas Nusi, Poernomo Boedi Setiawan, Herry Purbayu, Ulfa Kholili, Budi Widodo, Husin Thamrin, Amie Vidyani, Yudith Annisa Ayu Rezkitha, Yoshio Yamaoka

**Affiliations:** 1 Department of Internal Medicine, Dr. Soetomo Teaching Hospital, Faculty of Medicine Universitas Airlangga, Surabaya, Indonesia; 2 Division of Gastroentero-Hepatology, Department of Internal Medicine, Faculty of Medicine, Universitas Airlangga, Surabaya, Indonesia; 3 Helicobacter pylori and Microbiota Study Group, Institute Tropical Disease, Universitas Airlangga, Surabaya, Indonesia; 4 Department of Internal Medicine, Faculty of Medicine, University of Muhammadiyah Surabaya, Surabaya, Indonesia; 5 Department of Environmental and Preventive Medicine, Faculty of Medicine, Oita University, Yufu, Japan

**Keywords:** CRP, colitis, fecal calprotectin, inflammatory bowel disease

## Abstract

Inflammatory bowel disease (IBD) with a poor prognosis may be due to persistent colitis. According to the latest guidelines, monitoring has become a part of the treatment process for colitis. Adequate monitoring of the patient's condition is necessary to determine the course of the disease to prevent the worsening of the condition and suppress the subclinical inflammatory process. This analytical study with a cross-sectional design was conducted to evaluate the activity of colitis using the results of C-reactive protein (CRP) and fecal calprotectin (FC) assays. FC levels were analyzed by ELISA, while CRP levels were analyzed using Siemens Flex particle-enhanced turbidimetric immunoassay. In 30 subjects with endoscopy and biopsy of colitis, 16 men and 14 women had a median age of 52.5 (18–70) years. The median FC value increased by 67 (7.3–722 g/g) and was positive (≥50 g/g) in 20 subjects (66.7%), and the mean CRP value was 13.64 mg/L, positive (10–15 mg/L) in 13 subjects (43.33%), and negative (<10 mg/L) in 17 subjects (56.67%). This study demonstrated that FC had a significant relationship with CRP (r=0.57; p<0.001) in patients with colitis. Assessing the levels of FC and CRP among patients with colitis can be useful to assess the worsening of symptoms early and reduce mortality and morbidity.

## INTRODUCTION

Colitis can cause chronic diarrhea as one of its main symptoms. It is a condition in which the large intestine appears normal during colonoscopy examination, but the histological examination of the biopsy shows an increase in intraepithelial lymphocytes in the colonic mucosa. The main symptoms of people with colitis are lower gastrointestinal symptoms, namely chronic abdominal pain accompanied by diarrhea or constipation [1].

In developing countries, the incidence of colitis is increasing. Many factors influence the increase in the incidence, one of which is the lack of medical resources, which includes limited treatment [2]. Until now, the data used is still based on hospital reports (hospital-based), and in Indonesia, there has not been any epidemiological study on colitis. As of 2021, there were 10 cases of irritable bowel disease (IBD), 2.2–14.3 cases of ulcerative colitis (UC), and 3.1–14.6 cases of Chron's disease (CD) per 100,000 people in Indonesia. Based on data from the endoscopy unit of a hospital in Jakarta, Indonesia, in 2002, there were 5.2% of cases of Chron's disease (CD) and ulcerative colitis (UC), 12.2% cases of IBD with chronic diarrhea, hematochezia (3.9%), chronic diarrhea with bleeding and abdominal pain (25.9%), and only abdominal pain (2.8%).

The gold standard for diagnosing colitis is a combination of colonoscopy and anatomic pathology of the terminal ileum and large intestine to evaluate and classify the patient's risk using a simpler, non-invasive, and inexpensive investigation. With the progress of these supporting examinations, it is hoped to evaluate the course of the disease and monitor therapy success [3]. In inflammatory bowel disease (IBD), examination of fecal biomarkers to confirm the diagnosis and predict mucosal activity is no longer used because it can be variably elevated in some patients with microscopic colitis [4]. Therefore, the stool calprotectin (FC) examination is currently preferred as one of the mandatory tests for gastrointestinal inflammation, not only because it is more sensitive but also because it is non-invasive [5].

Biomarkers of intestinal mucosal inflammation include antibodies, acute phase proteins, and other proteins in serum or feces, but so far, there is no superior biomarker in microscopic colitis [6]. Although some literature suggests that microscopic colitis is associated with immune or inflammatory disease, the majority of patients do not show marked signs of systemic inflammation. The examination of general serological inflammatory markers usually reveals normal results or only a slight increase in collagenous colitis (CC) and lymphocytic colitis (LC). Therefore, the examination of general inflammatory markers does not significantly affect diagnostics but can still be used to monitor the course of the disease [4]. CRP is a common serological pro-inflammatory agent interacting with interleukin-6 (IL-6), interleukin-1β (IL-1β), and tumor necrosis factor-α (TNF-α). The developmental process of inflammation initiated by the disease agent stimulates the release of three essential cytokines, all of which induce CRP. The IL-6 cytokine mainly produced by macrophages and T cells can affect CRP levels because it directly induces acute-phase proteins released by the liver [7].

A study on IBD found that FC levels were 0.834 times correlated with increased endoscopic disease activity as measured using the Rachmilewitz index [8]. A similar study in ulcerative colitis using the modified Baron's index also found that FC correlated better with endoscopic disease activity than CRP (r=0.821 *vs*. 0.556) [9]. Based on the above studies, this study aimed to evaluate the relationship between FC and CRP as biomarkers in colitis and mucosal activity in IBD.

## MATERIAL AND METHODS

This observational study with a cross-sectional design was carried out after obtaining approval from the ethics committee of Dr. Soetomo Hospital and informed consent from the subjects.

### Study population

The sample size was calculated using the formula: n=[((Zα+Zβ))/(0.5 ln(1+r)/(1-r))]^2+ 3, using the correlation coefficient (r) from a previous study (-0.533) and 95% confidence interval. Subsequently, a minimum of 25 patients were required for the study. Patients included in the study were diagnosed with colitis, aged 18–70 years, visiting the gastroenterology clinic of RSUD, Dr. Soetomo Surabaya. Subjects with a history of gastrointestinal malignancy, decompensated cirrhosis, and chronic kidney disease were not included in this study. We collected data from the complete medical history, including age, weight, height, and general examination.

### FC levels

Fecal calprotectin (FC) levels were taken from the patient's stool preparations in an amount of at least 15 mg, which were analyzed with the PhiCal^©^ Calprotectin enzyme-linked immunosorbent assay (ELISA) kit (Immunodiagnostic AG, Stubenwald-Allee 8a, D-64625 Bensheim). The use of NSAIDs should be discontinued two days before the examination. The normal value of calprotectin is <50 µg/g.

### CRP

We assessed the levels of CRP from the patient's blood serum (3 mL) which was measured using the extended-range CRP examination at the Diagnosis Center Building, Dr. Soemoto Surabaya Hospital (Siemens Flex® Reagent Cartridge C-Reactive Protein, Frimley, Camberley, England) and then tested with the particle-enhanced turbidimetric immunoassay (PETIA) technique. Low-grade inflammation values used were 0.1 to 10–15 mg/l.

### Statistical analysis

Statistical analysis was performed using SPSS for Windows version 22.0, including univariate and bivariate analysis. Data analysis was performed using the Pearson correlation test (heterogeneous data) and the Spearman correlation test (homogeneous data). The normality test was performed using the Shapiro-Wilk test.

## RESULTS

This study was conducted at the gastroenterology clinic of RSUD, Dr. Soetomo Surabaya from March to August 2020. We collected data from 30 participants diagnosed with colitis through colonoscopy and anatomical pathology. The data obtained include gender, current symptoms, diagnosis based on pathological results, and medications received (Table 1). In this study, there were 16 (53.3%) male subjects and 14 (46.7%) female subjects. The most common symptom was diarrhea, 15 (50%) subjects complained of defecating with a liquid consistency more than three times a day with or without mucus and blood. Another symptom patients complained about was abdominal pain in 5 (16.67%) subjects. However, 10 participants (33.33%) did not have any complaints. The diagnosis of participants based on the results of anatomical pathology was dominated by non-specific chronic colitis in 28 (93.3%) subjects and only 2 (6.67%) other subjects diagnosed with colitis. Different types and doses of drugs were given to participants. 29 (96.7%) patients used sulfasalazine, while only 1 (0.33%) patient used lansoprazole. The administration of sulfasalazine ranged from 250 mg tablets twice daily to 500 mg three times daily. In Indonesia, sulfasalazine is available in 250 mg and 500 mg tablets, 4 g/60 mL enemas, and 500 mg suppositories. Based on the literature, the usable dose of 5-ASA is at least 3 grams per day. However, in this study, the dose used to achieve remission was 2–4 grams daily. The average remission is completed in 16–24 weeks, followed by a maintenance dose of 1.5–3 grams daily.

**Table 1:** General characteristics of the subjects.

Characteristic	N	%
**Gender**		
Female	14	46.67
Male	16	53.33
**Chief complaint**		
Diarrhea	15	50
Abdominal pain	5	16.67
No complaint	10	33.33
**Pathology**		
Unspecified colitis	28	93.33
Ulcerative colitis	2	6.67
**Treatment**		
Sulfasalazine	29	96.7
Lansoprazole	1	0.03

In this study, the data obtained were quite heterogeneous, as described in Table 2. The mean age of patients was 50.9±13.93 years, and the median was 52.5 years. The minimum age of patients was 18 years, and the maximum was 70 years. The mean level of fecal calprotectin (FC) was 118.11±145.65 µg/g with a median of 67 µg/g. The minimum value obtained was 7.3 µg/g, and the maximum was 722 µg/g. The mean FC levels in chronic non-specific colitis were 117.09±149.72 µg/g and 132.4±97.44 µg/g in ulcerative colitis. The results of FC examination were positive if 50 µg/g was found in 20 (66.67%) subjects and negative if <50 µg/g were found in 10 (43.80%) subjects. The mean level of C-reactive protein (CRP) was 13.64±17.48 mg/L, and the median was 5.25 mg/L. The minimum value obtained was 0.08 mg/L, and the maximum was 115 mg/L. Furthermore, the mean level of CRP in subjects with chronic non-specific colitis was 13.54±8.09 mg/L and 15±4.24 mg/L in ulcerative colitis. The results of the CRP examination were positive if 10–15 mg/L were found in 13 (43.33%) subjects and negative if <10 mg/L was found in 17 (56.67%) subjects.

**Table 2:** Characteristics of the subjects associated with FC and CRP.

Characteristic	N	%	Mean±SD	Median	Range
**Age (Years)**	-	-	50.9±13.93	52.5	18–70
**FC**					
Positive (≥50 µg/g)	20	66.7	118.11±145.65	67	7.3–722
Negative (<50 µg/g)	10	43.8			
**CRP**					
Positive (≥10 mg/L)	13	43.33	13.64±17.48	5.25	0.08–115
Negative (<10 µg/g)	17	56.67			

FC - Fecal Calprotectin; CRP - C-Reactive Protein.

The results of the Shapiro-Wilk normality test showed that FC and CRP data were normally distributed, so Pearson's parametric correlation statistical test was used to assess the relationship between FC and CRP levels. There was a significant relationship between FC and CRP with a correlation coefficient (r) of 0.57 (p-value=0.01). Furthermore, this relationship was significant even after adjusting for age (r=0.61, p=0.000). In addition, there was a significant relationship between FC and CRP levels (r=0.504, p-value=0.039) in the group of normal CRP levels (n=17). However, there was no significant relationship between FC and CRP levels (r=0.304, p-value=0.312) in the group with increased CRP levels (n=13).

## DISCUSSION

This study showed that in colitis patients, there was a significant correlation between FC and CRP levels, even after adjusting for age. FC levels have a better significance for detecting colitis compared to CRP. According to the literature, a minimum of two biomarkers, including FC and CRP, are required to establish a diagnosis of colitis in a patient. In a previous study, the normal value of fecal calprotectin used was 2 mg/L, with a test limit of 10 mg/L. The test limit for FC 10 mg/L is quite effective in identifying organic intestinal disease with a sensitivity of 89% and specificity of 79%, while FC levels greater than 100 g/g can detect active inflammatory bowel disease. Recent literature recommends an upper limit for FC levels at 50 g/g [10]. Another study examining CRP showed that at a serum concentration of 5 mg/l, it had high specificity for detecting IBD activity with endoscopic and biopsy confirmation. However, CRP has such a low sensitivity that a negative test result cannot exclude active inflammation [11].

Based on statistical analysis, it is known that the distribution of CRP data is not normally distributed. This can be seen from the average and maximum value of CRP, which is far above the cut-off (>10 mg/L). In non-specific chronic colitis, CRP examination is less effective in establishing a diagnosis without the help of other examinations, although it shows significant results in ulcerative colitis. In previous studies, data also showed that common serological inflammatory markers such as CRP did not show significant diagnostic results [4].

The POCER study on 86 asymptomatic colitis patients analyzed the relationship between FC and CRP levels with the severity of postoperative endoscopic recurrence [4]. Based on the Rutgeerts score, to detect recurrence by endoscopy examination of FC and CRP levels, ileocolonoscopy should be performed regularly within 18 months of resection. If there is a recurrence, examination of FC levels will give significant results. The limit for FC levels to differentiate the two conditions is 100 g/g (95% sensitivity, 54% specificity, 69% PPV, and 93% NPV) [12].

The secondary results of this study were that more than 50% of the subjects were male. In a previous study, the overall prevalence of microscopic colitis was 103.0 per 100,000 persons, including 39.3 per 100,000 for collagenous colitis and 63.7 per 100,000 for lymphocytic colitis without significant sex differences [13]. In this study, data on the age of the subjects were quite heterogeneous, with a minimum age of 18 years and a maximum age of 70 years. The elderly group (>60 years) was more susceptible to microscopic colitis. A previous study found that women are 20 times more prone to collagen colitis than men, whereas, in lymphocytic colitis, there was no significant difference between men and women [14].

In this study, half of the subjects experienced symptoms of diarrhea, namely defecation with a liquid consistency continuously three or more times in one day with or without mucus and blood. In addition, some subjects also complained of abdominal pain. Based on the literature, the appearance of additional symptoms, including abdominal pain, bloating, fatigue, and weight loss, can be associated with microscopic colitis, which can occur in up to 50% of cases [15]. In a previous retrospective study, the use of the mesalazine drug (5-aminosalicylic acid/5-ASA) improved the condition in 50% of cases. Several other therapies have also been tested, but no results can be used as a reference. In this study of 30 subjects without specific groupings, 1 subject did not receive treatment, while the other 29 subjects received sulfasalazine therapy with individual doses ranging from 250 mg tablets twice daily to 500 mg three times a day.

Our study is the first cross-sectional study aimed at evaluating biomarkers in patients with a diagnosis of colitis at the teaching hospital of Dr. Soetomo Surabaya, Indonesia. Limitations in this study include that there is no specific grouping of subjects based on disease staging, the absence of a complete stool examination, and stool culture so that it cannot rule out other factors that cause an increase in FC, such as infection.

## CONCLUSION

Fecal calprotectin (FC) has good diagnostic sensitivity and is significantly associated with C-reactive protein (CRP) in patients with colitis.

## Data Availability

Further data is available from the corresponding author on reasonable request.
